# Chick neonatal environment: the presence of a hen, not hatching system, promotes cecal microbiota development and induces limited long-term effects on immunity and redox balance

**DOI:** 10.1016/j.psj.2026.107536

**Published:** 2026-07-30

**Authors:** Sylvie Combes, Alice Hondelatte, Adrien Castinel, Nathalie Couroussé, Estelle Cailleau-Audouin, Pascal Chartrin, Estelle Godet, Claire Bonnefous, Anne Collin, Catherine Schouler, Karine Germain, Laurence A. Guilloteau

**Affiliations:** a GenPhySE, Université de Toulouse, INRAE, ENVT, 31326, Castanet-Tolosan, France; b INRAE, EASM, 17700, Surgères, France; c GeT‐PlaGe, Genotoul, INRAE, 31326, Castanet-Tolosan, France; d INRAE, Université de Tours, BOA, 37380, Nouzilly, France; e INRAE, Université de Tours, ISP, 37380, Nouzilly, France

**Keywords:** Chick-adult contact, On-farm hatching system, Gut microbiota, Immunity, Chicken

## Abstract

This study investigated how hatching environment and early-life exposure to an adult hen influence gut microbiota development, immune function, and redox status in broiler chicks. Male chicks were hatched either on-farm (**OFH**) or in a conventional hatchery (**CH**), with or without the presence of an adult hen for the first 15 days post-hatching (**CH+H, OFH+H**). An additional group received antibiotic treatment during this period (**CH+AB**). At 27 days post-hatching, the chicks faced suboptimal conditions (transport, temperature fluctuations, and increased density) and were vaccinated against Gumboro disease to test their resilience. Cecal bacterial gene communities and immune gene expression in the bursa of Fabricius and cecal tonsils were assessed at 20 and 56 days of age. Plasma immune, metabolic, and redox status were evaluated at 27, 40, and 56 days. At 20 days, hen contact promoted the development of the cecal microbiota, characterized by increased relative abundances of *Bacteroides, Prevotellaceae UCG-001, Parabacteroides, Mucispirillum, Desulfovibrio, Succinatimonas, Olsenella*, and *Sutterella*. In OFH chicks, hen contact reduced cecal IgA concentrations. Conversely, in CH+AB chickens, the bacterial diversity diminished, favoring *Escherichia–Shigella* dominance, and *TNF* gene expression was increased in the cecal tonsils. While the hatching system alone had no effect on microbiota, it acted synergistically with hen contact and further enhanced microbiota maturity. At 56 days, most early observed differences in microbiota composition had disappeared. However, in CH+H chickens, *IgA* gene expression decreased in the bursa of Fabricius and *CD40* mRNA expression increased in the cecal tonsils. In OFH+H chickens, the lipid oxidative status was reduced in favor of a better redox status compared to OFH chickens. In conclusion, the contact of chicks with a hen during the start-up period had a transient but significant effect on gut microbiota development during the initial colonization window. Although most effects diminished over time, lasting changes in immune function and redox status warrant further investigation to uncover the long-term health benefits of neonatal environmental enrichment in poultry.

## Introduction

The health, welfare, and performance of farm animals are highly dependent on the gut microbiome (Chen et al., 2021). Indeed, the intestinal microbiota is in constant interaction with different physiological compartments, including the digestive system, the immune system, and other organs such as the brain and liver, and it is influenced by their functioning and vice versa (Beldowska et al., 2023). In addition, the intestinal microbiota forms a physical and competitive barrier to pathogens, has a trophic role in the body, and is essential for shaping the immune system associated with the intestinal mucosa (Broom and Kogut, 2018). In poultry, the cecum is important in metabolism and immune maturation. Bacterial fermentation within the cecum provides up to 10% of a chicken’s metabolizable energy (Jozefiak et al., 2004). Tonsil development on the cecum, which belongs to the gut-associated lymphoid tissues, depends on the cecal microbiota. Moreover, the bursa of Fabricius close to the cecum is the primary lymphoid tissue for B lymphocyte production (Olah and Vervelde, 2008).

Naturally, the chick intestinal tract after hatching is exposed to the external environment and colonized by microbiological communities coming from the hen, conspecifics, and the environment. In commercial poultry production, broiler chicks hatch in incubators of hatcheries without the presence of the hen and are exposed to stressful conditions during the perinatal period, which impact the microbiota (Mandal et al., 2020) and induce immediate and long-lasting metabolic changes (Beauclercq et al., 2019; Foury et al., 2020), with consequences on chicken performance and welfare (de Jong et al., 2017). The gut microbiota composition is also influenced by the host’s genetics, age, sex, and diet (Cui et al., 2021; Yue et al., 2024). It can be modified by antibiotic treatment at taxonomic, metagenomic, and metabolomic levels (Wisselink 2017; Broom, 2018b; Plata et al., 2022).

To improve the early perinatal conditions of chicks, alternative on-farm hatching systems provide the chicks with immediate access to feed and water ad libitum and limit the exposure to stressors encountered in conventional hatcheries (de Jong et al., 2019). The effects of on-farm hatching systems on broiler health, welfare, and performance are quite beneficial (de Jong et al., 2020; Giersberg et al., 2021), as well as on intestinal health (Akram et al., 2025). The contact of chicks with an adult hen at hatching also provides a donor of microbiota (Kubasova et al., 2019). Early transmission of adult microbiota to the chicks accelerates the maturation of their immune system (Meijerink et al., 2020; Zenner et al., 2021). Guilloteau et al. (2024) analyzed the benefits/risks of on-farm hatching system combined with the presence or absence of an adult hen on hatchability, chick quality scores, performance, health, and robustness in suboptimal rearing conditions. The study highlighted the benefits of on-farm hatching for chick health and performance. The presence of the hen during the chick start-up phase interacted with hatching conditions and the sex of the chicks.

In this study, we analyzed the effects of hatching systems and the presence of the hen during the start-up phase described by Guilloteau et al. (2024) on the gut microbiota, the immune response in the cecal tonsils and bursa of Fabricius, and plasma metabolites in male broiler chickens. The combination of conventional hatching treatment with antibiotics was added as an experimental group of antibiotic treatment. Our hypothesis is that on-farm hatching of chicks and their contact with an adult hen facilitate the establishment of a microbiota and immune system maturation that would help in maintaining health and performance.

## Materials and methods

### Ethics approval

All experimental procedures were approved by the Ethics Committee COMETHEA POITOU-CHARENTES n°84 (APAFIS#24474-2020021816237418 v3) and carried out following current European Union legislation (EU Directive 2010/63/EU).

### Experimental design

**Fig. 1:**
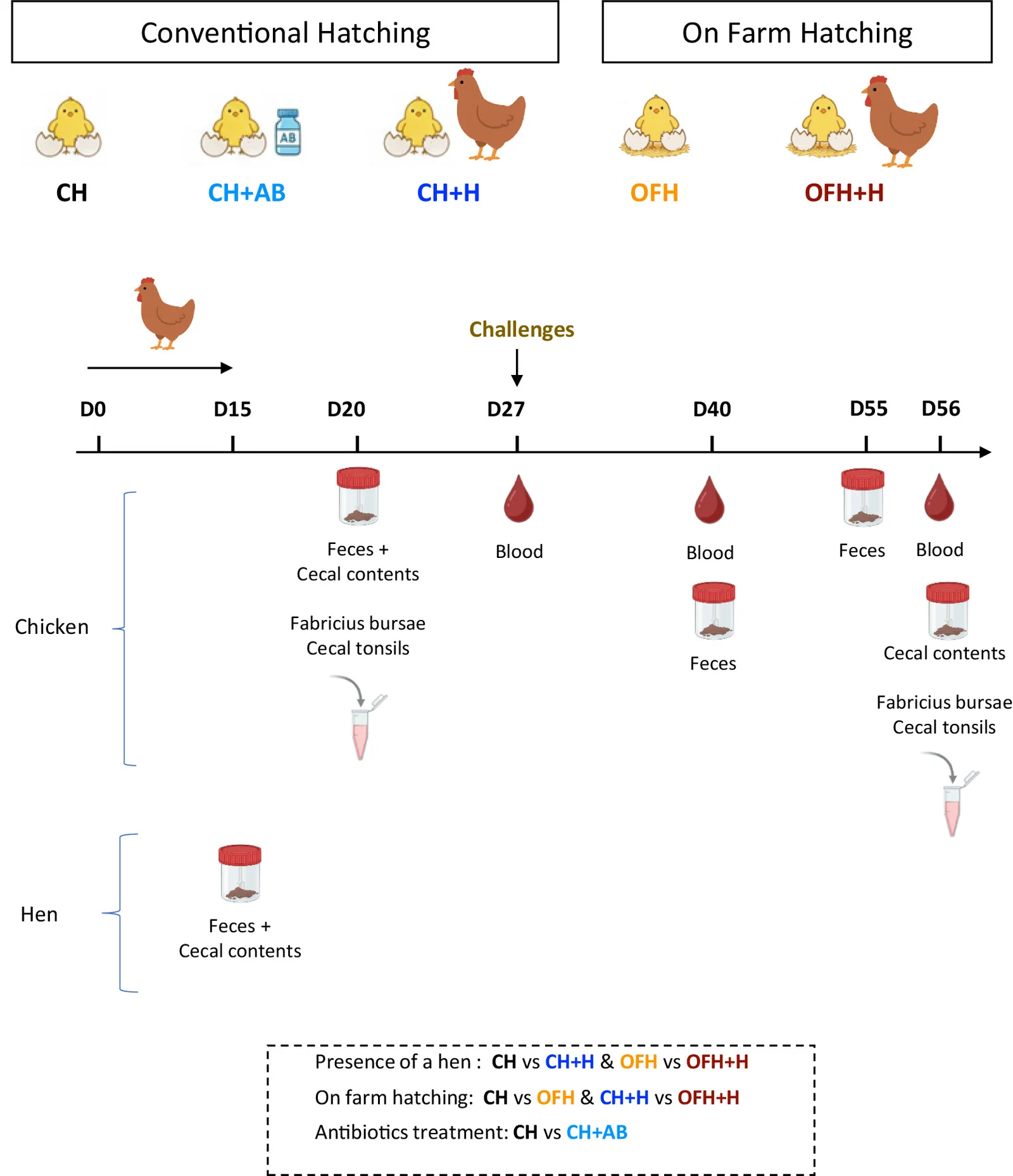
Experimental design. Chicks were allocated into five experimental groups: conventional hatchery (CH) or on-farm hatching (OFH), with or without the presence of an adult hen (CH+H and OFH+H), and treatment with antibiotics after hatching at the hatchery (CH+AB).

The experimentation consisted of combining different hatching conditions, chicks with or without hens, and with or without antibiotic treatment. These factors were integrated in a multifactorial design for all conditions. A total of 720 one-d-old certified JA 757 (males and females), among which 432 were from a conventional hatchery (**CH**) and 288 were hatched on-farm (**OFH**), were allocated into five experimental groups as described by Guilloteau et al. (2024): 1) CH with no hen; 2) CH + antibiotic treatment (**CH+AB**); 3) CH + hen (**CH+H**); 4) OFH with no hen; 5) OFH + hen (**OFH+H**). The CH+AB group was included in order to create a group with the establishment dynamic of a perturbed microbiota. The experimental design and tissue sampling schedule are shown in Fig. 1.

### Hatching and rearing conditions

The chicks were placed in 3-m^2^ pens (18 chicks/pen) that were randomly placed in the room; each experimental group had eight replicate pens. The antibiotic treatment, ADJUSOL® TMP SULF Liquid (25 mg/kg of sulfadiazine and 5 mg/kg oftrimethoprim, Virbac, Carros, France) for 5 d starting on Day (D) 2 and SURAMOX 50 (20 mg/kg of amoxicillin, Virbac) for 5 d on D19–D23, was only applied in the chicks’ drinking water for the CH+AB group. These medications are commonly used antibiotics and dosages applied for digestive and respiratory diseases. Chick sex was determined on D19 at the end of the start-up phase, and the number of chicks was adjusted to a maximum of 16 per pen, keeping a balanced ratio between males and females. On D27, the chicks were exposed to suboptimal rearing conditions that may be encountered on farms: transport in boxes at a lower temperature and fasting for 4 h; high chick density; and vaccination with an infectious bursal disease virus (**IBDV**) live vaccine (Gumboro vaccine) (HIPRAGUMBORO® - G97, Hipra France, Saint-Herblain, France) in the drinking water. The chicks had *ad libitum* access to water and feed without any anticoccidial drugs. They were fed with a standard starter diet (raw energy = 4462 kcal/kg) until D19, then a grower diet from D20 to D34 (4527 kcal/kg) and a finisher diet from D35 to D56 (4600 kcal/kg) (Supplemental Table S1) (Arrivé Nutrition Animale, Saint-Fulgent, France). A wire mesh platform (101 × 50 cm) and a perch (length = 55 cm) were used for environmental enrichment.

As the effects of the hens’ presence at hatching and during the chick start-up phase on performance interacted with the hatching condition and the sex of the chicks, we chose to characterize the microbiota, immunity, and physiological responses only in males because CH+H male chicks were more impacted by the presence of hens compared to CH chicks, whereas CH+H females were not, and OFH+H females were positively impacted by the presence of the hen (Guilloteau et al., 2024). As the number of chicks was adjusted to a maximum of 16 per pen on D19, the number of male chicks sampled on D20 was variable (8–14 per rearing condition) to maintain 16 chicks per rearing condition on D56. Bursa of Fabricius, cecal tonsils, and cecum contents were collected at D20 and D56, at the end of the start-up and finishing phases, respectively. Feces and blood samples were collected on the same birds at D27 before suboptimal rearing conditions were applied, 13 days later (D40), and at the end of finishing phase (D56). Tissues were weighed and stored at -80°C for subsequent RNA extraction. Feces and cecal contents were stored at -80°C for microbiota analyses. Blood was sampled using 5-ml heparin vacutainer tubes, and plasma was collected after centrifugation and stored at -80°C for biochemical analyses.

### Starting period of chicks in contact with hens

Sixteen Lohmann Brown hens, acting as natural gut microbiota donors and adult presence for chicks, were obtained from a local commercial egg-laying hen farm. The hens were aged 31 weeks, vaccinated according to the standard vaccination program, under controlled sanitary conditions, and declared free of *Mycoplasma gallisepticum, Mycoplasma synoviae, Chlamydia psittaci*, and *Salmonella pullorum gallinarum*. Only Ascaris and Heterakis parasites were detected at a very low level in hen feces. In the experimental room, the hens were placed separately in wire-latticed pens (3 m^2^) with a nest box, perch, and feed and water *ad libitum*. Hens were accustomed to their new environment for 12 days, fed with a standard rearing diet for laying hens (30099G25, Arrivé Nutrition Animale, Saint-Fulgent, France), and allowed to deposit fecal and cecal materials (and thus microbiota) on litter. A wire-latticed space (101 × 50 cm) for chicks was placed in their pen two days before chick arrival or egg hatching. Embryonated, 18-d-old eggs were laid under infrared heat lamps for OFH. Eight hens were used for eight groups of 18 OFH chicks, and eight hens were used for eight groups of 18 CH chicks. On D0, 1-day-old CH chicks were placed in the pen’s wire-latticed space; OFH chicks were already in this space. The procedure used to put in contact hens and chicks is described by Guilloteau et al. (2024). Free in-access feed and water were placed under the wire-latticed space for chicks (not accessible to hens), and in raised troughs for hens (not accessible to chicks). Chicks were able to leave and exit the wire-latticed space at will. Hens were present with the chicks for 2 weeks, the critical period for chick start, and removed on D15. Weight and clinical examinations of the hens were recorded the day before they were installed in the pens. Hens’ feces and cecal contents were collected on D15.

### 16S rRNA gene amplicon sequencing and sequence analysis of cecal content and feces

DNA was extracted from 25–30 mg of cecal or fecal content using the DNA Mini Kit (Qiagen) according to the manufacturer instructions and optimized by three lysis steps and an additional purification step. PCR amplicons of the 16S rRNA gene V3-V4 region (forward primer 3’-ACGGRAGGCAGCAG-5’, reverse primer 3’-TACCAGGGTATCTAATCCT-5’) were sequenced by MiSeq technology (Illumina) at the Genomic and Transcriptomic Platform (GeT-PlaGe, Institut National de Recherche pour l'Agriculture, l'Alimentation et l'Environnement (**INRAE**), Toulouse), as described by Read et al. (2019). Sequencing reads were deposited in the National Center for Biotechnology Information Sequence Read Archive (accession number: PRJNA1365110). Amplicon sequences were analyzed by the FROGS version 4.1 (Find Rapidly OTUs with Galaxy Solution pipeline) (Escudié et al., 2018), following the guidelines. Merged sequences (350–500 nucleotides) were clustered into amplicon sequence variants (**ASV**) by using Swarm (clustering aggregation distance: 1). After PCR chimera removal using vsearch, the ASV present in less than three samples or that represented less than 0.005% of all sequences were filtered out (Bokulich et al., 2013). The taxonomic affiliation of the ASV was performed with the 16S SILVA database (138.1, pintail 100, Quast et al., 2013). The mean number of reads per sample was 19028 (range 7283–35464). For α and β-diversity analyses, the count table was rarefied to 7283 sequences per sample.

Microbiota richness (number of observed ASV), Shannon index, and Inverse Simpson (**invSimpson**) α-diversity index were calculated using the phyloseq R package (MacMurdie and Holmes, 2013). The unrarefied count table was used to assess the relative abundances of bacterial taxa at the phylum, family, and genus level.

### Gene expression profiling in the bursa of Fabricius and cecal tonsils

Frozen tissues were ground into powder. A 100-mg sample was transferred to screwap microtubes containing 1 mL of Invitrogen TRIzol reagent (12044977, Fisher Scientific, Illkirch-Graffenstaden, France) and a metal bead and vigorously shaken for 5 min on ice. Total RNA was extracted in accordance with Désert et al., 2016. Ten μL of 5 N acetic acid were added with chloroform to reduce DNA contamination. The quality of total RNA was assessed using RNA Nano chips on a 2100 Bioanalyzer Instrument (Agilent, Waldbronn, Germany). All samples had an RNA Integrity Number score >8.0. The total RNA concentration was measured with a UV-Vis spectrophotometer (Nano Drop 1000 Spectrophotometer, Thermo Fisher Scientific, Illkirch-Graffenstaden, France) at 260/280 nm absorbance and RNA integrity was confirmed by agarose gel electrophoresis (1%) with the presence of the two major ribosomal RNA bands (28 S and 18 S). A DNase treatment using a DNA-free DNA removal kit (AM1906, Thermo Fisher Diagnostics, Villebon sur Yvette, France) was performed to avoid DNA contamination.

An aliquot of 2 μg of total RNA was reverse-transcribed in cDNA with Superscript IITM (200U, 18064-022, Thermo Fisher Scientific) and random primers. Real-time qPCR was carried out in a LightCycler® 480 (Roche Diagnostics, Meylan, France). Primer sequences are presented in Table S2. Real-time qPCR analyses were conducted using 7.5 μL Takyon No ROX SYBR MasterMix blue dTTP (UF-NSMT B0701, Eurogentec, Seraing, Belgium) containing each primer (10μM) and 2.5 μL of cDNA. Target genes were amplified using the following thermocycler program: 95°C for 5 min, followed by 45 PCR cycles at 95°C for 10 s and 60°C for 20 s, and a final melting curve step at 72°C for 10 s. The efficiency of amplification was established for each primer pair by utilizing the serial dilutions of cDNA, and each sample was run in triplicate. Glyceraldehyde-3-phosphate dehydrogenase (*GAPDH*), hypoxanthine-guanine phosphoribosyltransferase (*HPRT1*) and TATA binding protein (*TBP*) genes were selected as reference genes for bursa of Fabricius, and *HPRT1, TBP*, and eukaryotic translation initiation factor 3 Subunit F (*EIF3F*) genes were used as reference genes for cecal tonsils. The quantification of PCR reactions for each primer pair was carried out by comparing the target gene with the reference genes for which a normalization factor had been calculated using the GeNormsoftware (Microsoft Excel GeNormalgorithm, version 3.5, Vandesompele et al., 2002) to establish the relative gene expression. Gene expression of the target gene was calculated using the following equation (Pfaffl, 2001; Vandesompele et al., 2002):

E = 2^Ct reference - Ct sample/normalization factor

where E is gene expression, Ct is the cycle threshold and Ct reference is the mixture of all cDNA samples.

### Immunoglobulins in the cecal content and blood plasma

The cecal contents were diluted at 100 mg/mL in Tris Buffered Saline and centrifuged to collect the supernatant. Protein concentration was determined using the Bradford method (Quick Start™ Bradford 1x Dye Reagent #5000205, Bio-Rad, Marnes la Coquette, France). IgA titers were defined with ELISA kits (BETE33-103, Bethyl Laboratories, FortisR Life Sciences, Boston, MA). Titers are expressed as ng/mg protein.

IgM, IgY, and IgA were detected in blood plasma by indirect ELISA kits (BETE33-102; BETE33-103; BETE33-104, Bethyl Laboratories, FortisR Life Sciences, Boston, MA). Antibody titers are expressed in ng/mL.

### Immune response to vaccine

Immune system activity was assessed by measuring the antibody titers specific to IBDV that were present in the plasma of the chicken before vaccination (D27), 13 days (D40) and 29 days later (D56). Antibody titers were determined by ELISA using the ID Screen® IBD VP2 Indirect kit and the protocol described by the supplier (ID.vet, Grabels, France).

Antibody titers were defined as:

log10 (titer) = 0.97 × log10 (S/P) + 3.8; S/P = (DO_sample_ - DO_negative control_)/(DO_positive control_ - DO_negative control_)

Titer = 10^log10 (titer). Chickens were considered positive for antibody response to IBDV when the antibody titer was >1324.

### Physiological parameters of the blood

The metabolic, antioxidant, and oxidative status of the blood and inflammation parameters were measured. Commercial kits (Thermo Fisher Diagnostics) were used to determine plasma glucose (mg/L) (MG981780), uric acid (mg/L) (MG981788), and triglycerides (mg/L) (MG981786). Total plasma antioxidant activity was determined through total antioxidant status (**TAS**) (mmol/L) (NX 2332, Randox Laboratories, Roissy, France). Protocols were used in accordance with supplier instructions and adapted to an automated Thermo Scientific Arena 20XT photometric analyzer (Thermo Fisher Diagnostics). Haptoglobin-like activity described as PIT54 in chicken (Wicher et al., 2006) was evaluated using a colorimetric assay (TP-801, Tridelta Development Ltd, Maynooth, Ireland) measuring inhibition of hemoglobin peroxidase activity and expressed as mg/mL. Lipid peroxidation was determined using spectrophotometry of thiobarbituric acid reacting substances (**TBARS**) as described by Travel et al. (2021) and expressed as nM/mL.

### Statistical analysis

All statistical analyses were carried out using R software, version 4.3.0 in RStudio software. β-diversity was investigated by Bray-Curtis distance and visualized with non-metric multidimensional scaling (**nMDS**). To check hen and chick group differences, an ADONIS pairwise test with the Bray-Curtis distance was carried out. To assess the dynamics of community evolution and the proximity of an adult hen, the Bray-Curtis distances between the microbiota of hens and chicks were evaluated in each group and across both age groups. To evaluate the degree of similarity between the chicks and the hen with which they were in contact, the Bray-Curtis distance between them was compared with the Bray-Curtis distance between chicks and hens from the other pens. All the aforementioned group distance comparisons were analyzed using a non-parametric two-sample Wilcoxon test with a Benjamini-Hochberg (**BH**) *P* value correction. The α-diversity index, fourth root transformed microbiota taxonomic data with a threshold of 0.5% of the relative abundance for quantitative repeatability (Paës et al., 2020), and log transformed data collected at slaughter were analyzed using a mixed effect linear model with treatment, age, and their interaction as fixed effects and pen as a random effect. Due to variance heterogeneity among age groups, treatment effect was further analyzed for each age group using a one‑way analysis of variance. To account for multiple testing, *P* values were adjusted using the Hochberg methods for microbiota taxonomic data. For time series data (blood parameters), a fourth root transformation was performed. These transformed data were analyzed using a linear mixed model with treatment, age, and their interaction as fixed effects and pen and individual as random effects. Treatment effect was further analyzed within each age group by using a one‑way analysis of variance with treatment effect. Multiple pairwise comparisons between treatments were performed using a Tukey adjustment. A significance threshold was set at *P* < 0.05.

## Results

### Effect of neonatal enrichment on cecal microbiota

**Fig. 2:**
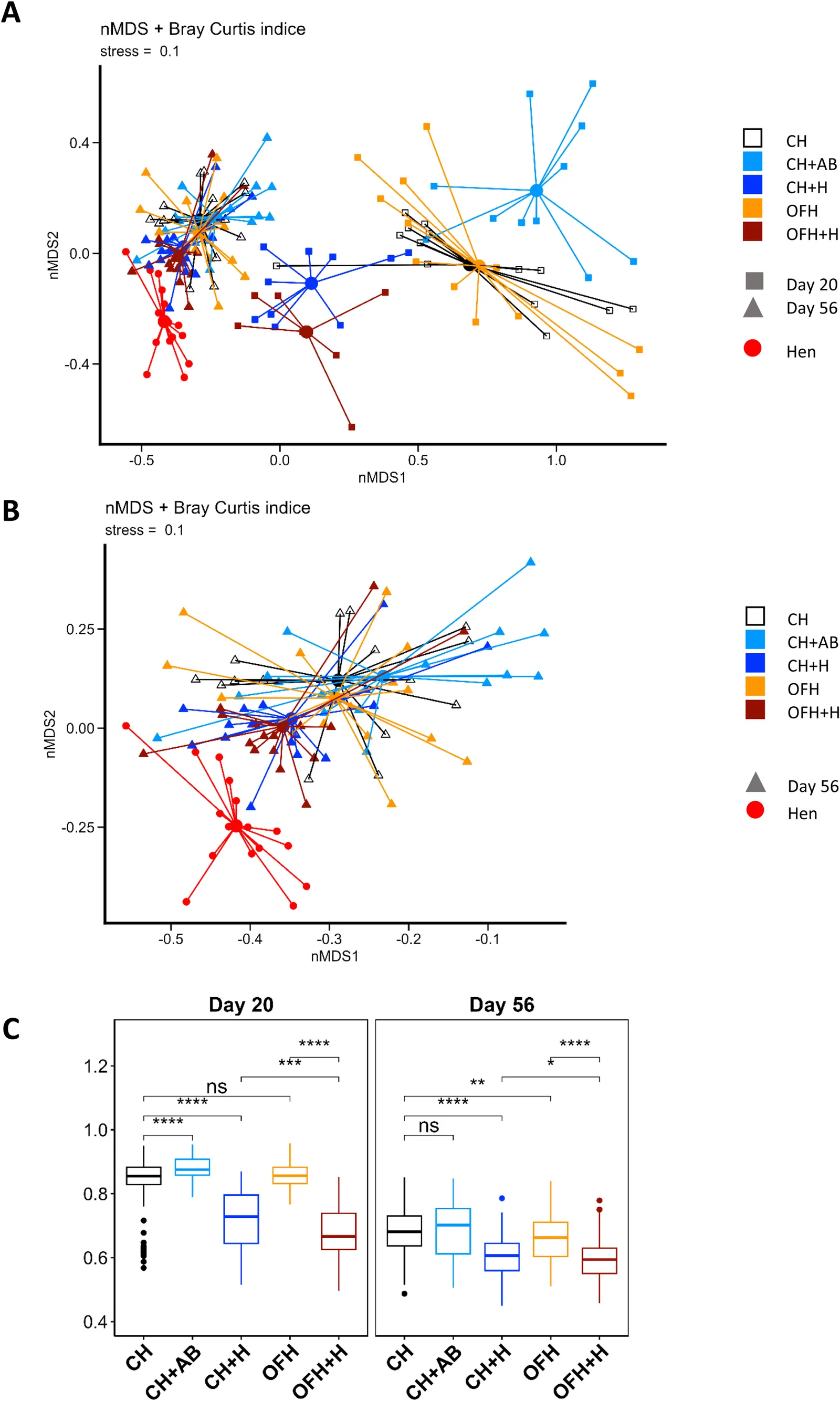
Cecal microbiota β-diversity of hen and chicks according to hatching conditions with or without the presence of an adult hen. (A) Non-metric distance scaling (nMDS) analysis of cecal microbiota at 20 and 56 days of age and of the adult hens (B) and focus on 56 days of age and the adult hens. (C) Bray-Curtis distances between the microbiota of hens and chickens in each group and across both age groups. “*” to “****” correspond to significant differences between treatment groups. Sample sizes at day 20: CH n = 14; CH+AB n = 11; CH+H n = 10; OFH n = 13; OFH+H n = 7; at day 56, n = 16 broilers per group; hen n = 16.

Given the high variability observed in the feces microbial community structure (Supplemental Fig. S1), we chose to focus our analysis on the cecal compartment. A shift and lower dispersion of the cecal bacterial community structure of chicks was observed with age, which approached similarity to that of hens (Fig. 2A, Supplemental Fig. S1). Richness (Observed ASV), Shannon index, and invSimpson index significantly increased between 20 and 56 d post-hatching (*P* < 0.05) to reach values similar to those of hens (Supplemental Fig. S1).

**Fig. 3:**
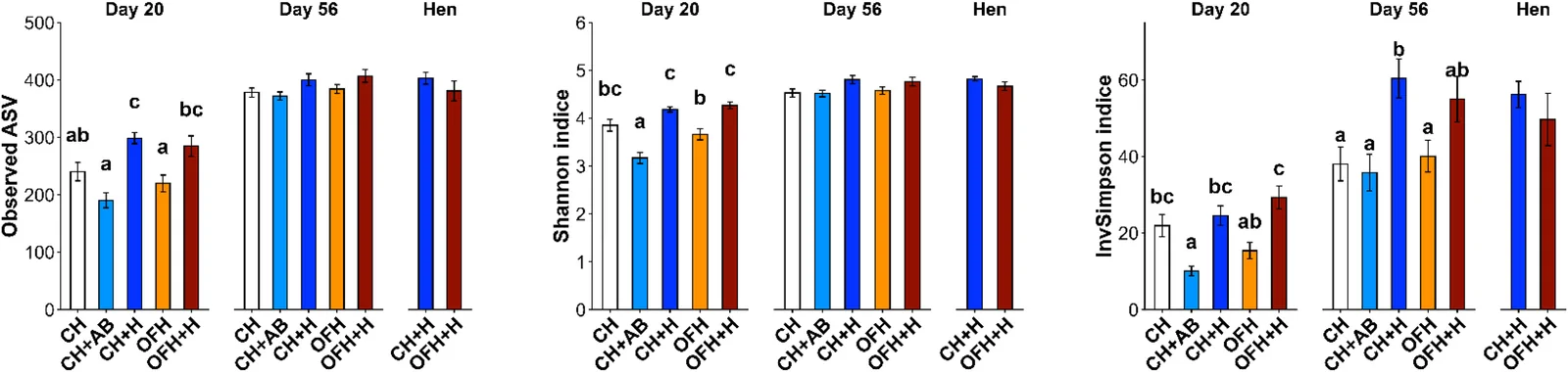
Alpha-diversity indexes. Chicks were allocated into experimental five groups: conventional hatchery (CH) or on-farm hatching (OFH), with or without the presence of an adult hen (CH+H and OFH+H), and treatment with antibiotics after hatching at the hatchery (CH+AB). Values are expressed as means ± SE; different letters correspond to significant differences (*P* < 0.05) among groups. Sample sizes at day 20: CH n = 14; CH+AB n = 11; CH+H n = 10; OFH n = 13; OFH+H n = 7; at day 56, n = 16 broilers per group; hen n = 8.

***Contact with a hen improved cecal microbiota dynamics.*** Compared to chicks raised without a hen, the presence of a hen altered the bacterial community structure in CH and OFH chicks by D20, with a more pronounced effect observed in OFH chicks (13 % vs. 21 % of explained group distance variance, Table 1). Richness, Shannon index, and InvSimpson index demonstrated an increase in diversity among the 20-day-old chicks that were in contact with a hen (Fig. 3). These increases were significant for all three indices in OFH chicks, whereas only richness was significantly increased in CH chicks. At D56, only the invSimpson index was higher in the CH+H chicks than in the CH group. Furthermore, at D20 and D56, the bacterial communities of chicks that had contact with a hen were more similar to the hen's microbiota than those of chicks that had no contact with a hen (Fig. 2A, B). The Bray Curtis distance between hen and chick bacterial communities was significantly shorter when they were in contact compared to chicks not sharing space with a hen (CH+H vs. CH; OFH+H vs. OFH; *P* < 0.001; Fig. 2A-C).

**Table 1:** Explained variance (R²) and adjusted *P* value of PERMANOVA pairwise comparison of Bray Curtis distance within groups and age of chicken cecal microbiota according to the hatching system and/or the presence adult hen at hatching.

Groups^1^		CH		CH+H		OFH	
	Age	Day 20 n = 14	Day 56 n = 16	Day 20 n = 10	Day 56 n = 16	Day 20 n = 13	Day 56 n = 16
CH+AB	Day 20 n = 11	0.152 *P* = 0.001					
	Day 56 n = 16		0.035 NS				
CH+H	Day 20 n = 10	0.134 *P* = 0.001					
	Day 56 n = 16		0.064 *P* = 0.007				
OFH	Day 20 n = 13	0.044 NS					
	Day 56 n = 16		0.053 *P* = 0.029				
OFH+H	Day 20 n = 7	0.077 NS		0.077 NS		0.207 *P* = 0.001	
	Day 56 n = 16		0.031 NS		0.031 NS		0.064 P = 0.005

NS: *P* value > 0.05

1 CH conventional hatchery-hatched chicks; OFH: on-farm hatched chicks, CH+H: conventional hatchery-hatched chicks with the presence of an adult hen, OFH+H: on-farm hatched chicks with the presence of an adult hen, CH+AB: antibiotics treated chicks after hatching at the conventional hatchery.

The microbiota of chicks was closer to that of the hens with which they were in contact than with the microbiota of hens in the other pens but only at D56 (Supplemental Fig. S2A). ASV analysis showed that 20-day-old chicks in contact with a hen for 15 days shared 10% of their ASV with the hens (CH+H: 64 out of 649 ASV; OFH+H: 55 out of 580 ASV; Supplemental Fig. S2B). These ASV were absent in chicks without hen contact. At D56, when the chicken microbiota was closer to that of the hens in all groups, specifically shared ASV between hens and chicks that were in contact with hens were no longer evident. This result indicates a potential transfer of microbiota from adult hens to chicks during the 15 days the latter were in contact with a hen and was observable at least until 20 days of age.

**Fig. 4:**
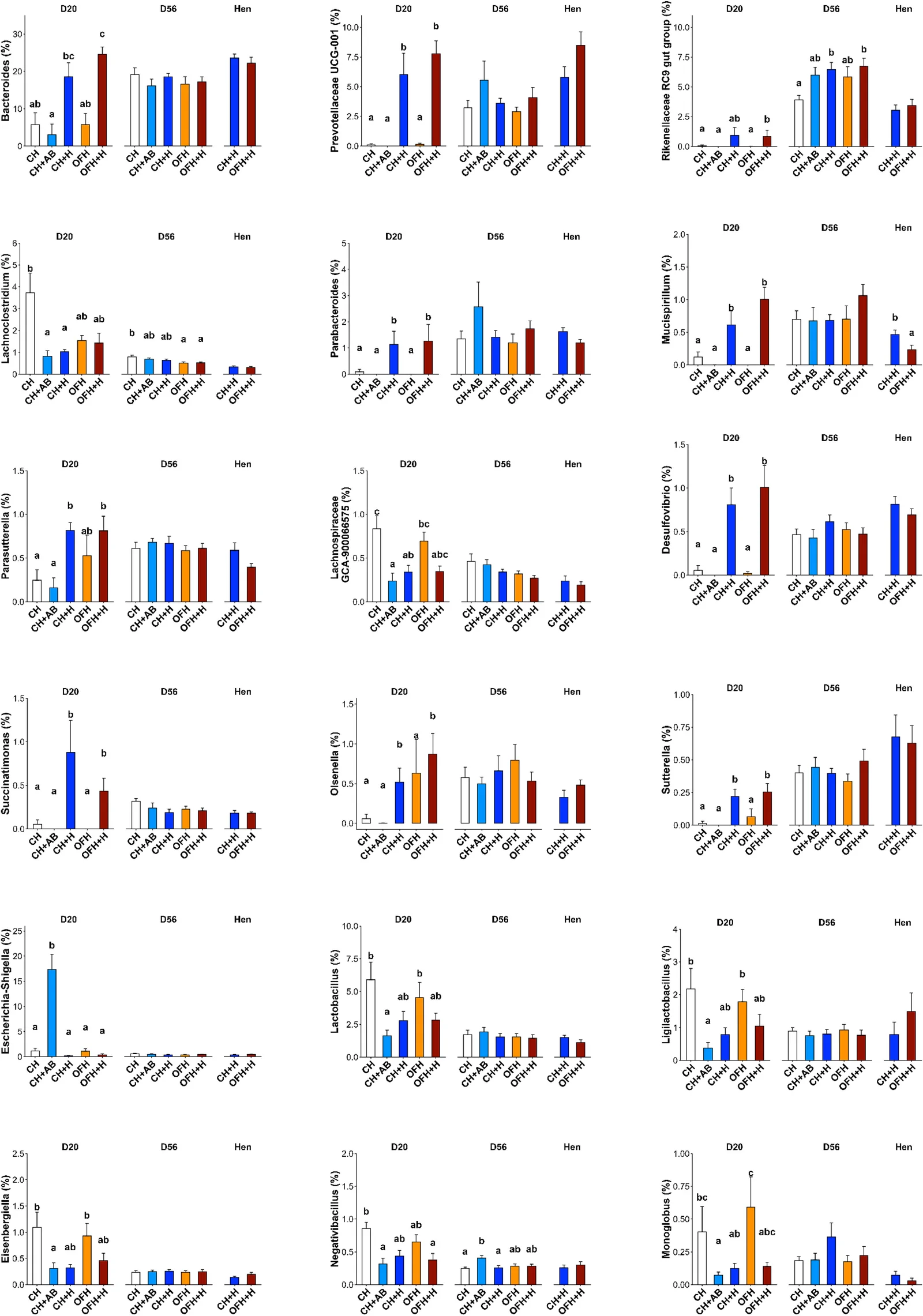
Relative abundance of bacterial genera in the cecum according to hatching conditions. Chicks were allocated into experimental five groups: conventional hatchery (CH) or on-farm hatching (OFH), with or without the presence of an adult hen (CH+H and OFH+H), and treatment with antibiotics after hatching at the hatchery (CH+AB). (Hen: cecal bacterial genus relative abundance of the adult hens in CH+H and OFH+H groups). Values are expressed as means ± SE; different letters correspond to significant differences (*P* < 0.05) among groups. Sample sizes at day 20: CH n = 14; CH+AB n = 11; CH+H n = 10; OFH n = 13; OFH+H n = 7; at day 56, n = 16 broilers per group; hen n = 8.

The bacterial composition of the hens in the CH+H and OFH+H groups did not differ significantly (Supplemental Fig. S3, Supplemental Table S3). The presence of a hen profoundly altered the composition of the cecal microbiota in 20-day-old chicks. However, this effect was no longer or sporadically (one minor genus, *Ruminococcaceae DTU089*) observed at 56 days of age (Table S3). In 20-day-old chicks in contact with a hen (CH+H, OFH+H), the taxonomic composition at the phylum level showed higher relative abundance of *Bacteroidota, Deferribacterota*, and *Desulfobacterota* compared to their respective control groups reared without a hen, along with a significant decrease in *Firmicutes* (*P* < 0.05; Supplemental Fig. S3, Supplemental Table S3). At the genus level (Fig. 4, Supplemental Table S3), the presence of a hen significantly increased the relative abundance of *Prevotellaceae UCG-001, Parabacteroides, Mucispirillum, Desulfovibrio, Succinatimonas, Olsenella*, and *Sutterella* (*P* < 0.05) in 20-days-old chicks. *Bacteroides* and *Rikenellaceae RC9 gut group* increased only in OFH+H chicks, whereas *Parasutterella* increased exclusively in CH chicks (*P* < 0.05).

Taken together, these results indicate that the presence of an adult hen improved the chick cecal microbiota development observed at 20 and 56 days of age, profoundly altering its taxonomic composition at least until 20 days of age.

***On Farm Hatching Slightly improved Cecal Microbiota dynamics.*** No significant differences were detected at D20 and D56 in the pairwise comparisons of cecal microbiota community structure between OFH and CH chicks nor between OFH+H and CH+H chicks (Fig. 2A, B, Table 1). The α-diversity index also did not significantly vary between the two hatching processes (Fig. 3). Microbiota maturity, namely the distance to reach the hen microbiota structure, of the OFH+H group was significantly more proximal to the hen microbiota than in the CH+H group at D20 (*P* < 0.001, Fig. 2C). At D56, the microbiota of OFH chicken exhibited closer proximity to the microbiota of adult hens, irrespective of the presence (OFH+H) or absence of a hen (OFH) (Fig. 2C). Hatching process did not affect the taxonomic composition of the chick microbiota at the phylum level nor at the level of well-defined genera (Supplemental Table S3). Altogether, these results suggest that on-farm hatching improve microbiota dynamics and had a synergetic effect with hen contact.

***Antibiotic Treatment Delayed Microbiota Dynamics in Early Life*.** At D20, β-diversity analysis revealed that antibiotic treatment explained 15 % of the variance of the CH+AB vs. CH between-group Bray-Curtis distance (Fig. 2A, Table 1). Antibiotic-treated chicks displayed reduced microbiota diversity, as indicated by significantly lower Shannon and InvSimpson indices compared to untreated chicks at 20 days of age (*P* < 0.05, (Fig. 3). The antibiotic-treated chicks exhibited a greater Bray-Curtis distance from the hen microbiota than the CH chicks, which suggests delayed microbiota maturation (Fig. 2A, C). This reduction in diversity in antibiotic-treated chicks may be attributed to a dominance of *Escherichia-Shigella* in their microbiota, along with a decreased relative abundance of seven other genera (*Lachnoclostrium, Lachnospiraceae GCA-9000066575, Lactobacillus, Ligilactobacillus, Eisenbergiella, Negativibacillus*, and *Monoglobus*; Fig. 4)*.* However, by D56, the effects of antibiotic treatment on bacterial community structure (Fig. 2B, Table 1), α-diversity (Fig. 3), maturity (proximity to the hen bacterial community structure, Fig. 2C), and taxonomic composition (Supplemental Fig. S3; Table S3) of the cecal microbiota were no longer observed, indicating no lasting effect of antibiotic treatment after the challenge.

### Effect of hen presence and hatching conditions on immune response, metabolism, and redox balance

The presence of hens with OFH chicks reduced the chicken body weight on D20 before the IBDV vaccination and environmental challenges (Table 2). Although the mean ceca weight in CH+H and CH+AB groups was greater than the mean ceca weight in CH group before the challenges (D20), the mean weights of the cecal tonsils and bursa of Fabricius did not significantly differ among the groups before and after the challenges (Table 2). Moreover, no group effect was observed on IBDV-specific antibody titers before and after vaccination nor between titers of negative and positive chicks (Supplemental Table S3; Supplemental Fig. S4).

**Table 2:** Organ weight of chicken according to age and the hatching system and/or the presence adult hen at hatching.

Groups^1^	CH	CH+AB	CH+H	OFH	OFH+H	*P* value
Day 20						
n	14	11	10	13	8	
BW (g)	561.7 ± 9.4^ab^	557.9 ± 9.7^ab^	547 ± 12^ab^	583 ± 12^b^	519 ± 25^a^	0.031
Empty ceca / BW	0.35 ± 0.02^a^	0.45 ± 0.02^b^	0.44 ± 0.02^b^	0.40 ± 0.02^ab^	0.47 ± 0.03^b^	<0.001
Cecal tonsils / BW	0.043 ± 0.003	0.041 ± 0.003	0.047 ± 0.003	0.044 ± 0.004	0.056 ± 0.006	0.090
Fabricius bursa / BW	0.289 ± 0.017	0.318 ± 0.018	0.273 ± 0.017	0.276 ± 0.016	0.291 ± 0.024	0.392
Day 56						
n	16	16	16	16	16	
BW (g)	2727 ± 58	2738 ± 67	2646 ± 67	2845 ± 71	2830 ± 69	0.224
Empty ceca /BW	0.312 ± 0.01^b^	0.287 ± 0.009^ab^	0.312 ± 0.01^b^	0.285 ± 0.008^ab^	0.271 ± 0.013^a^	0.014
Cecal tonsils/BW	0.03 ± 0.002	0.034 ± 0.002	0.033 ± 0.002	0.033 ± 0.002	0.028 ± 0.002	0.144
Fabricius bursa / BW	0.124 ± 0.016	0.139 ± 0.015	0.15 ± 0.013	0.158 ± 0.016	0.123 ± 0.016	0.281

1 CH: conventional hatchery-hatched chicks; OFH: on-farm hatched chicks, CH+H: conventional hatchery-hatched chicks with the presence of an adult hen, OFH+H: on-farm hatched chicks with the presence of an adult hen, CH+AB: antibiotics treated chicks after hatching at the conventional hatchery. Values are expressed as means ± standard error; *P* value < 0.05 and different letters correspond to significant differences between treatment groups.

**Fig. 5:**
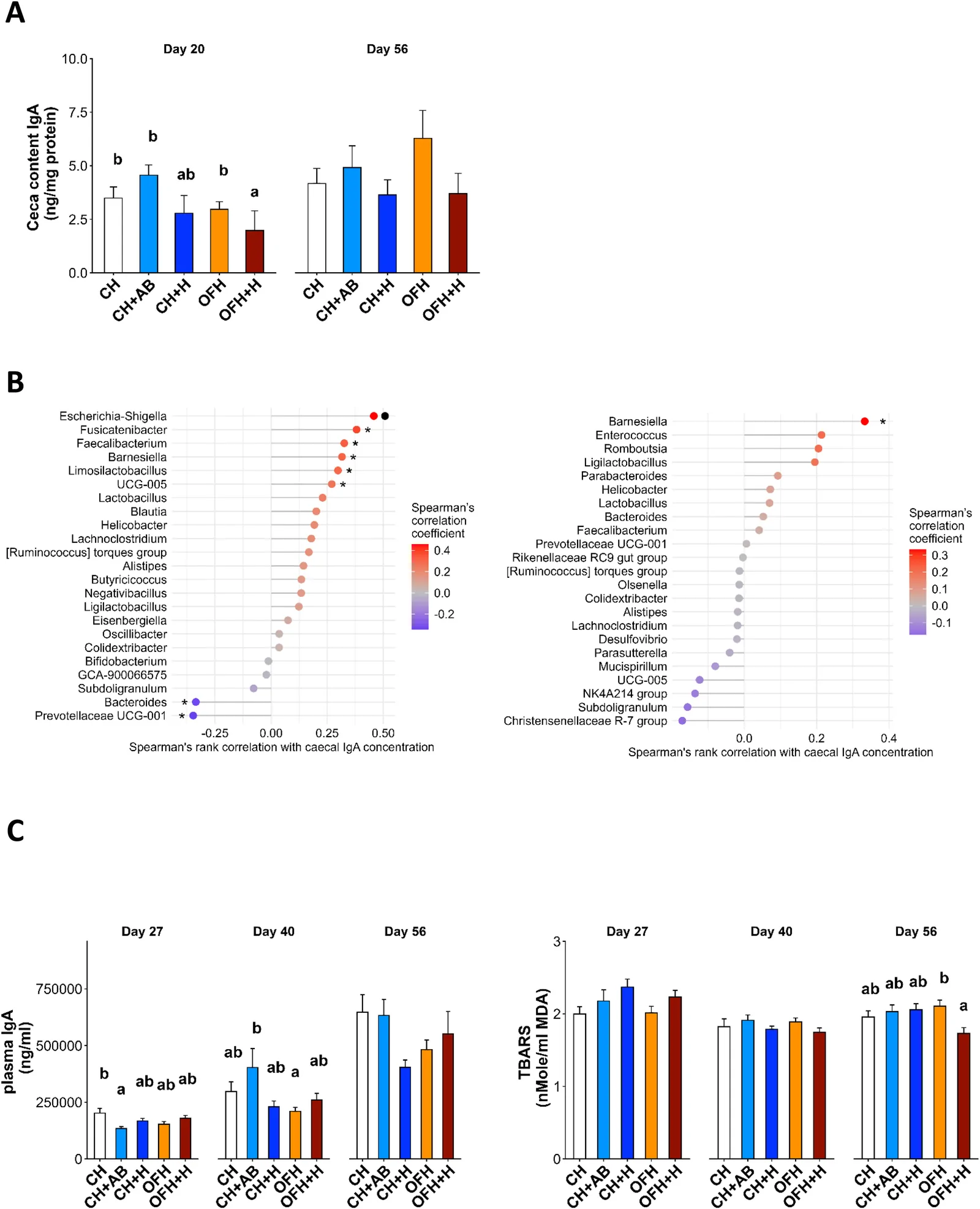
IgA concentration in the cecum and blood plasma according to chick hatching process. (A) Cecal IgA content. (B) Correlation between cecal IgA concentration and cecal bacterial genus relative abundance. (C) Plasma IgA concentration and thiobarbituric acid reacting substances (TBARS) values. Chicks were allocated into five experimental groups: conventional hatchery (CH) or on-farm hatching (OFH), with or without the presence of an adult hen (CH+H and OFH+H), and treatment with antibiotics after hatching at the hatchery (CH+AB). Values are expressed as means ± SE; “*” and different letters correspond to significant differences (*P* < 0.05) among treatment groups. Sample sizes at day 20: CH n = 14; CH+AB n = 11; CH+H n = 10; OFH n = 13; OFH+H n = 7; at day 56, n = 16 broilers per group. For plasma IgA and TBARS, n =16 chicks per group.

Before the challenges, OFH+H group had significantly reduced cecal IgA concentrations by D20 (OFH+H vs OFH; *P* < 0.05; Fig. 5A). This difference was not observed after the challenges (D56). Interestingly, bacterial taxonomic modification could be correlated with IgA cecal concentration at least at 20 days of age. By D20, cecal IgA concentrations were significantly positively correlated with *Escherichia-Shigella* relative abundance after BH correction (Fig. 5B). Although correlations with the abundance of seven other genera were observed, they were no longer significant after applying BH correction. Antibiotic treatment significantly decreased plasma IgA concentration before the challenges (CH+AB vs. CH; *P* < 0.05 Fig. 5C), but not after (D40 and D56).

**Fig. 6:**
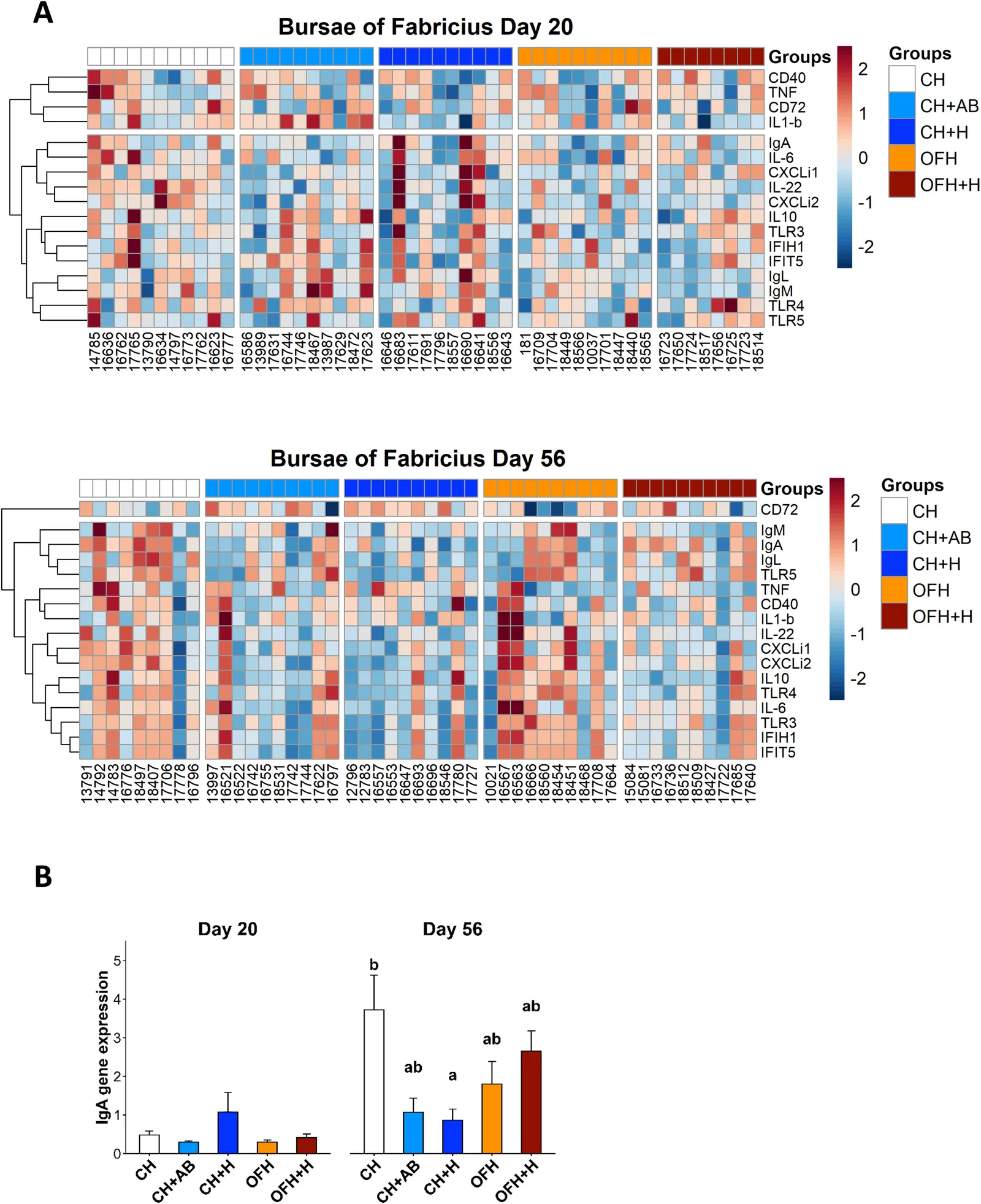
Gene expression in the bursa of Fabricius at days 20 and 56 in chicks hatched in a conventional hatchery (CH) or on-farm hatching (OFH), with or without the presence of an adult hen (CH+H, OFH+H), and in hatchery-hatched chicks treated with antibiotics (CH+AB). (A) Heatmap representation of gene expression: columns represent individual samples and rows represent genes. Expression levels are color-coded (red = high, blue = low). The left dendrogram clusters genes with similar expression patterns. (B) Barplot of IgA gene expression in bursa of Fabricius. n = 8–11 chicks per group and per age.

**Fig. 7:**
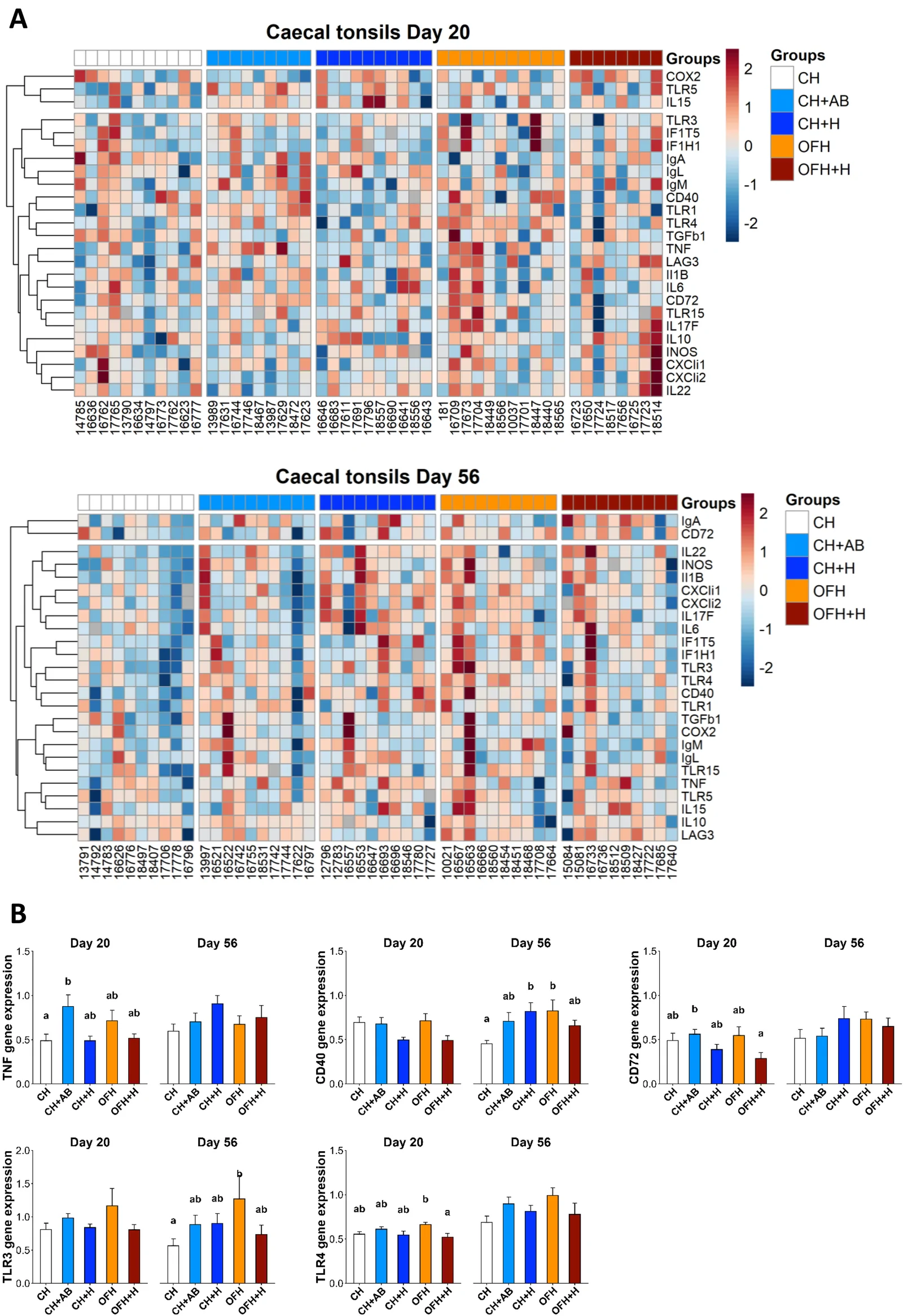
Gene expression in the cecal tonsils at days 20 and 56 in chicks hatched in a conventional hatchery (CH) or on-farm hatching (OFH), with or without the presence of an adult hen (CH+H, OFH+H), and in hatchery-hatched chicks treated with antibiotics (CH+AB). (A) Heatmap representation of gene expression: columns represent individual samples and rows represent genes. Expression levels are color-coded (red = high, blue = low). The left dendrogram clusters genes with similar expression patterns. (B) Barplot of gene expression in cecal tonsils with differential expression between groups. n = 8–11 chicks per group and per age.

Neonatal enrichment with OFH and the presence of the hens modified the expression of a limited number of genes in the bursa of Fabricius and cecal tonsils (Fig. 6, Fig. 7). Before the challenges on D20, *TLR4* gene expression was reduced in the cecal tonsils of OFH+H chickens compared to OFH chickens, and *TNF* gene expression increased in the cecal tonsils of CH+AB chickens. On D56, *IgA* gene expression was significantly reduced in the bursa of Fabricius, and *CD40* gene expression increased in the cecal tonsils of the CH+H group compared to the CH group (Fig. 6B), although there was no significant difference in IgA concentration in the cecal content between these two groups (Fig. 5A). *TLR3* and *CD40* gene expression in the cecal tonsils was higher in OFH chickens than in CH chickens at D56 (Fig. 7B). Overall, the environmental and vaccination challenges did not influence plasma metabolism and redox balance among groups except for TBARS, which was significantly lower in OFH+H chickens than in OFH chickens (P < 0.05 Fig. 5C; Supplemental Table S3).

Altaken together, these results showed that hatching conditions and the presence of hens had little impact on gene expression in lymphoid tissues associated with the intestine and on metabolism of chickens even after the challenges.

## Discussion

The neonatal environment has a profound and lasting influence on the health, welfare, and performance of chickens. The bacterial environment of chicks at hatching is limited to the microbial communities present in the incubators of hatcheries and in the litter upon their arrival in the facilities. In this study, we hypothesized that enriching the neonatal environment of chicks through contact with an adult hen and by OFH could promote microbiota establishment and immune system maturation, thereby supporting health and performance. The effects of the rearing conditions on performance were reported by Guilloteau et al. (2024). Our results showed that contact with a hen after hatching promotes the development of the chick’s cecal microbiota, whereas OFH had little effect compared to CH system.

The bacterial colonization of chicks begins at hatching (Ballou et al., 2016; Ding et al., 2017; Volf et al., 2021), although several studies report possible vertical transfer of microbiota *in ovo* (Lee et al., 2019) and horizontal trans-shell migration (Van Veelen et al., 2018). Upon hatching, the chicks are exposed to a variety of microbes that form a metacommunity. A random sample of bacteria from this metacommunity with high dispersal capabilities and rapid growth generally colonizes ecological niches first and further facilitates the success of later colonizers (Connell and Slatyer, 1977; Christian et al., 2015). The characteristics of the newborn's microbiota community are, therefore influenced by the diversity, relative abundance, and spatial distribution of the colonizing bacteria in the metacommunity (Curtis and Sloan, 2004; Teyssier et al., 2018). We demonstrated that enrichment of the neonatal environment with the presence of a hen significantly impacted the microbial community assembly of chicks. As previously observed (Li et al., 2022), the diversity and similarity of the chick cecal microbiota to that of the hen increased compared to chicks raised without an adult. In our study, contact with a hen for 15 days shifted the chicks’ microbiota towards a higher proportion of commensal bacteria also present in the hen, including the genera *Bacteroides, Parabacteroides, Parasutterella, Mucispirillum, Desulfovibrio, Succinatimonas, Olsenella*, and *Sutterella*. An increase in the relative abundance of *Bacteroides, Parabacteroides, Mucispirillum, Desulfovibrio*, and *Olsenella* has been previously observed when chicks were in contact with a hen (Kubasová et al., 2019).

Overall, our results are consistent with the literature and support the importance of the presence of a hen as a reservoir of bacterial diversity available for colonization of the chick's intestine early in life. The manipulation of gut colonization in chicks early in life, through competitive exclusion using complex communities derived from adult individuals to compensate for the absence of maternal contact, is a well-studied concept (Impey et al., 1982; Kubasová et al., 2019, Zenner et al., 2021). While these studies generally report an inflection in the colonization process, the bacterial species favored vary among experiments. Three factors may explain these discrepancies: (1) environmental influences; (2) the initial composition and rapid temporal changes of the initial gut microbiota in young chicks; and (3) the stochastic nature of bacterial implantation from the metacommunity, the composition of which remains only partially characterized. *A priori*, hen-associated environmental exposure of broiler chicks would not control the aforementioned sources of variability in microbial transmission but could provide social interactions that likely contribute to chick welfare (Edgar et al., 2016).

Despite the strong effect of hen contact on microbiota establishment in 20-day-old chicks, immune parameters and oxidative status were only slightly affected by this modification of the early life environment. However, our results showed a decrease of *TLR4* expression in the cecal tonsils and a decrease in IgA secretion in the cecal content of OFH+H group. Interestingly, we observed a positive correlation between cecal IgA concentrations and *Escherichia-Shigella* relative abundance in 20-days-old chickens. This positive correlation may suggest a response to opportunistic bacterial colonization through an antigen-specific, T cell-dependent pathway. Therefore, the reduced IgA production in OFH+H chickens could reflect a lower colonization by potential pathogens, favoring commensal bacteria and reducing the stimulation of T cell-dependent IgA production. However, IgA is also known to play a role in maintaining and promoting commensal bacteria with lower growth rate within biofilms. Further studies are thus needed to elucidate the mechanisms of IgA action in the context of our study to better understand the significance of the observed reduction in cecal IgA concentration in chicks reared with a hen.

OFH had only a minor effect on the chicks’ microbiota and no effect on immune parameters compared to CH chicks at D20. In agreement, Akram et al. (2024, 2025) reported that OFH had no effect on microbiota composition but enhanced bursa of Fabricius development and increased duodenal villus width. Although overall gene expression profiles did not differ substantially, *IL-6, IFN-γ*, and *AVBD9* were specifically up-regulated in OFH chicks compared to CH chicks at D38 in Ross chickens (Akram et al., 2025). We observed an up-regulation of *CD40* and *TLR3* gene expression in the cecal tonsils of OFH chickens compared to CH chicks at D56, suggesting a better activation of innate (Diaz-Beneitez et al., 2025) and adaptative (Zhang et al., 2024) immune responses. The limited differences observed between the two hatching systems are noteworthy, as greater divergences were expected. Chicks hatched in hatcheries experience prolonged feed deprivation and transport and might already be colonized by early pioneer bacterial species from the hatchery before their arrival in the rearing facilities, which could adversely affect subsequent microbiota establishment. Conversely, OFH system allows immediate access to feed and water, potentially fostering a more favorable gut environment for the establishment of microbial communities. According to Akram et al. (2024), other factors intrinsic to the host, such as body weight, may act as stronger drivers in shaping the intestinal microbiota, thereby overshadowing the effects of hatching conditions but not the effect of contact with a hen. Our results suggest, however, a synergistic effect of hatching conditions and hen contact on chick cecal microbiota maturity. Indeed, in contact with hens, chick microbiota maturity, assessed by its similarity to the adult microbial structure, was greater in OFH chicks than in CH chicks. It is possible that OFH enriches the metacommunity available for intestinal colonization in chicks with additional environmental bacteria (in this case from building). Such additional bacterial species may already be present in the hens’ microbiota, thereby increasing the likelihood of successful colonization. Alternatively, environmental bacteria may act as facilitators that promote the establishment of the developing bacterial community (Curtis and Sloan, 2004).

In our experimental design, we also included a group of chicks that received an antibiotic treatment at therapeutic doses commonly prescribed in broiler production to control digestive and respiratory diseases. This group was considered to represent a perturbed microbiota dynamic. At D20 post-hatching, antibiotic treatment modified the bacterial community structure, reduced microbial diversity compared to the control, likely due to the dominance of *Escherichia–Shigella*, and increased *TNF* gene expression in the cecal tonsil, reflecting inflammation. Sulfadiazine and trimethoprim have been widely used since the 1970s to treat enteric infections caused by *Salmonella, Shigella*, and enterotoxigenic *Escherichia coli* (Masters et al., 2003). In our study, *Escherichia-Shigella* species appeared favored at the expense of commensal bacteria such as *Lachnoclostridium, Lactobacillus, Ligilactobacillus, Eisenbergiella, Negativibacillus*, and *Monoglobus*, possibly because of resistance to the antibiotic treatment*.* A decline in *Lactobacillus* spp. following amoxicillin treatment has previously been reported (Kerek et al., 2025). These taxonomic shifts observed in antibiotic-treated chicks resulted in greater Bray-Curtis distance from the highly diverse hen microbiota than the conventional non-treated chickens, suggesting delayed microbiota maturation.

The comparison of the five experimental groups supports the hypothesis that the presence of a hen restores the natural trajectory of microbiota establishment in chicks. If the presence of a hen is considered the baseline condition for this species, the absence of maternal contact may delay the normal development of the gut’s microbiota. Under this interpretation, the beneficial effect of hen contact would be a restoration of the species-specific developmental trajectory that would naturally occur under maternal care in addition to social interactions and environmental enrichment.

Interestingly, most of the effects of the experimental factors (i.e., hen contact, hatching system, and antibiotic treatment) on microbiota structure and taxonomic composition were no longer observed at slaughter on D56. Accordingly, in the study by Li et al. (2022) conducted with laying hens, the increase in α-diversity in chicks raised in the presence of a hen was only detectable up to 7–11 days of age. Two hypotheses can be proposed to explain the temporary effect. (1) The improvement of cecal microbiota maturation, particularly in hen-associated chicks, may occur only during the initial colonization phase and establishment of pioneer species. These pioneer species might primarily act as facilitators, promoting the installation of a microbiota that is ultimately shaped and optimized to its environment. (2) Vaccination and environmental stress may exert a stronger leveling effect on microbiota structure than our neonatal enrichment practices. As the gut microbiota can be considered as a complex adaptive system, it is one of the host’s fastest-adjusting components to environmental changes (Laforest-Lapointe and Arrieta, 2017). Thus, chicken microbiota composition might represent a unified, global response to the applied challenge experienced by the host. However, the absence of long-term effects of neonatal enrichment on microbiota composition does not exclude possible lasting effects on other physiological parameters, such as immune functions and redox balance. At D56, after the challenges, we observed a decrease in *IgA* expression in the bursa of Fabricius and in plasmatic IgA secretion, as well as an increase in *CD40* expression in the cecal tonsils in CH+H chickens. In addition, a reduction in lipid oxidative status (TBARS) was detected in the plasma of OFH+H chickens. These immune-related changes could be more closely associated with the response to the IBDV vaccine (Li et al., 2018; Zhang et al., 2024) than with the neonatal enrichment itself. However, the benefit for the chicks to be in contact with hen microbiota during the start-up period remains intact. The health status of the hens must be carefully controlled to ensure that no pathogens might be transmitted to the chicks. The use of mother hens may strengthen the bond with the chicks and possibly facilitate the transfer of the microbiota to the chicks.

### Conclusion

Our study demonstrated that enriching the neonatal environment of chicks through contact with a hen significantly improved the maturation of the cecal microbiota by promoting the establishment of a community structure more similar to that of the adult. In contrast, OFH alone had a limited effect on microbial composition, immunity parameters, and oxidative status compared to CH. However, a synergistic interaction between hen contact and OFH was observed, suggesting that the combination of both factors may enhance microbial maturity during early life. Antibiotic treatment, used here as a reference for microbiota disruption, delayed microbiota establishment and markedly reduced bacterial diversity and favored the proliferation of *Escherichia-Shigella* dominance at the expense of commensal genera, confirming the sensitivity of early microbial assembly to antibiotic medications. Hatching process and the presence of a hen had only a transient effect on microbiota composition at 20 days of age, since differences disappeared by 56 days of age, indicating that the influence of early environmental enrichment may be restricted to the initial colonization window. Nonetheless, such early microbial modulation could have longer-term physiological consequences. Overall, our findings highlight the critical role of early-life microbial exposure in shaping the microbiota development in chicks. While the observed effects on immune parameters were limited under the present experimental conditions, future studies conducted on commercial chicken farms by adapting neonatal microbial enrichment would better reveal the potential of overcoming biosecurity risks and the benefits of robustness in poultry.

## Funding

This research was supported by a grant from INRAE, Department of Animal Physiology and Livestock Systems for the RIMEL network.

## Data access

All raw sequences were deposited in the NCBI Sequence Read Archive (accession number PRJNA1365110)

Supplemental material

Table S1. Feed formulation of chickens during starter (D1-D19), grower (D20-D34), and finisher (D35-D56) periods.

Table S2. Sequence of the primers used for gene expression profiling in the bursa of Fabricius and cecal tonsils.

Table S3. Relative abundance of cecal bacterial phyla, families, and genera in chicks and hens and blood plasma parameters. Chicks were allocated into five experimental groups: conventional hatchery (CH) or on-farm hatching (OFH), with or without the presence of an adult hen (CH+H, OFH+H), and hatchery-hatched chicks treated with antibiotics (CH+AB).

Supplemental Figure S1. Cecal bacterial diversity according to age in chicks and hens.

Supplemental Figure S2. Chick cecal microbiota similarity with hen cecal microbiota. (A) Bray-Curtis distances between the chicks and the hen with which they were in contact. The distance between them was compared (Within) with the distance between chicks and hens from other pens (Between). (B) Shared amplicon sequence variants between chicks and hens. Chickens were allocated into five experimental groups: conventional hatchery (CH) or on-farm hatching (OFH), with or without the presence of an adult hen (CH+H, OFH+H), and hatchery-hatched chicks treated with antibiotics (CH+AB).

Supplemental Figure S3. Taxonomic characteristics of the cecal microbiota of chick and hens at the phylum (A), family (B), and genus (C) levels. Chicks were allocated into five experimental groups: conventional hatchery (CH) or on-farm hatching (OFH), with or without the presence of an adult hen (CH+H, OFH+H), and hatchery-hatched chicks treated with antibiotics (CH+AB).

Supplemental Figure S4. Positive and negative responses of chickens to infectious bursal disease virus vaccination according to experimental group: conventional hatchery (CH) or on-farm hatching (OFH), with or without the presence of an adult hen (CH+H, OFH+H), and hatchery-hatched chicks treated with antibiotics (CH+AB).

## Author contribution

LAG, KG and AC designed the study with the help of CS. LAG, CB, AC and CS performed the experiment. AH performed DNA extraction and PCR of cecal and fecal contents, and AC sequencing. NC, ECA, PC, and EG performed RNA extraction from tissues and PCR, immunoglobulin and physiological parameter analyses. SC analyzed microbiota data and LAG analyzed the others data. SC and LAG wrote the paper. All the authors reviewed and approved the manuscript.

## Disclosures

The authors declare they have no conflict of interest relating to the content of this article.

## Appendix Supplementary materials

Image, application 1

Image, application 2

Image, application 3

Image, application 4

## Footnotes

Supplementary material associated with this article can be found, in the online version, at doi:10.1016/j.psj.2026.107536.
